# Outbreak of hand, foot, and mouth disease caused by coxsackievirus A6 in a Juku in Fengtai District, Beijing, China, 2015

**DOI:** 10.1186/s40064-016-3307-x

**Published:** 2016-09-23

**Authors:** Jin-Song Li, Xiao-Gen Dong, Meng Qin, Hui-Ru Feng, Jun-Yong Yang, Ruo-Xi Li, Jian-Jun Zhang, Li-Shu Zheng

**Affiliations:** 1 Key Laboratory for Medical Virology, Ministry of Health, National Institute for Viral Diseases Control and Prevention, China CDC, 100 Ying-Xin St., Xi-Cheng District, Beijing, 100052 China; 2 Fengtai District Center for Diseases Control and Prevention of Beijing, 3 Xi An St., Feng-Tai District, Beijing, 100071 China

**Keywords:** Coxsackievirus A6, HFMD, Outbreak

## Abstract

An outbreak of hand, foot, and mouth disease (HFMD) that occurred in a Juku in Fengtai District, Beijing, China, in 2015 was monitored by the China Information System for Disease Control and Prevention. Epidemiological investigation showed that 11 cases occurred from two classes in the preschool art training department in the Juku. Coxsackievirus A6 (CV-A6) was identified as the causative pathogen of the outbreak via sequences analysis of products of real-time reverse-transcription polymerase chain reaction (RT-PCR) and nested RT-PCR. Phylogenetic analysis showed that CV-A6 strains isolated in this study clustered with epidemic strains isolated in China since 2013. The outbreak ended quickly with effective measures. This event indicates that continuous surveillance of HFMD etiological agents other than enterovirus 71 and coxsackievirus A16 is necessary.

## Background

Hand, foot, and mouth disease (HFMD) is characterized by mild febrile illness and vesicular exanthema on hands, feet and mouth. HFMD is a virus-induced infectious disease affecting mainly infants and children, typically causing outbreaks in kindergartens and primary schools. Severe cases can also involve serious complications, such as encephalitis, meningitis, or death. HMFD is considered a substantial public health threat, and ranked first among category C infectious diseases by the Ministry of Health of the People’s Republic of China in 2008. The predominant causative agents of HFMD are enterovirus 71 (EV-A71) and coxsackievirus A16 (CV-A16); additionally, other CVA, CVB, and some echoviruses have been reported to cause HFMD (He et al. 2013). Since 2012, a newly emerged enterovirus (CV-A6) has rapidly replaced the original circulating strains EV-A71 and CV-A16, becoming the predominant etiological agent of HFMD in some areas in China (Han et al. 2014; Lu et al. 2014). CV-A6 is classified as human enterovirus A in the family *Picornaviridae*, genus *Enterovirus*, and is an etiological agent of mild herpangina or HFMD (Mirand et al. 2012; Osterback et al. 2009; Sinclair et al. 2014; Wei et al. 2011). CV-A6 has also been reported to cause outbreaks worldwide (Feder et al. 2014; Puenpa et al. 2013; Fujimoto et al. 2012; He et al. 2013).

## Methods

### Study design

An HFMD outbreak associated with CV-A6 in a Juku in Fengtai District, Beijing, China was monitored by the China Information System for Disease Control and Prevention, From July 17–22. The outbreak was investigated immediately after it was reported to Fengtai District Center for Disease Control and Prevention (CDC) of Beijing on July 19 2015. Two investigators comprehensively recorded the entire investigation and completed all of the relevant documents appropriately.

### Samples and laboratory investigation

Samples were collected from the cases, close contacts and environmental surface in the classroom. Twelve pharyngeal swabs were collected from two teachers, two parents of the sick children, and eight sick children, in addition to five swab samples that were obtained from desks and a hand wash basin in the classroom. Additionally, three stool samples were collected from the other three sick children.

Suspensions of rectal swabs and swabs of environment surfaces were prepared by adding 1 ml phosphate-buffered saline (PBS; pH 7.2) and briefly vortexing. Suspensions of faecal samples were prepared by vortexing 0.2 g of faeces with 800 µl PBS for 3 min followed by centrifugation at 13,000 rpm for 10 min. Viral RNA was extracted using the QIAamp Viral RNA Mini Kit (QIAGEN, Hilden, Germany). All samples were tested for the presence of enterovirus, EV-A71 and CV-A16 by real-time RT-PCR using a real-time TaqMan reverse-transcription polymerase chain reaction (RT-PCR) kit from Jiangsu Shuoshi Bio-Tech Co., Ltd. (Hangzhou, China). cDNA from enterovirus-positive samples was synthesized using primer OL68-1 (GGTAA(C/T)TTCCACCACCA(A/T/G/C)CC). The resultant fragments, which included the partial 5′ untranslated region, VP2, and complete VP4, were amplified by nest RT-PCR with primers OL68-1 and MD91 (CCTCCGGCCCCTGAATGCGGCTAAT), followed by nest pair primers OL68-1 and EVP4 (CTACTTTGGGTGTCCGTGTT), as previously reported (Ishiko et al. 2002). The fragments were sequenced using the ABI 3730 system (Applied Biosystems, Foster City, CA, USA).

### Nucleotide sequence similarity and phylogenetic analysis

Virus genotype and nucleotide sequence similarities were analysed by Blastn online. Phylogenetic analysis based on the complete VP4 fragments of the most reference strains of CV-A6 in GenBank was conducted with the neighbor-joining method using the MEGA5.0 software program.

## Results

### Epidemiological investigation

A 5-year-old boy, who attended the preschool art training in a Juku in Fengtai District, Beijing, China, had a fever (38.4 °C) on July 17, and red vesicular lesions appeared the next day. From July 17–22, 11 cases in the Juku were reported by different hospitals to the China Information System for Disease Control and Prevention. Three cases were reported on July 17, and 4 cases each were reported on July 18 and 19. The 11 cases, which included 5 girls and 6 boys, were from two classes that shared noontime rest in the same room. Nine (81.8 %) cases exhibited vesicular exanthema on hands, feet and in the mouth, 9 cases (81.8 %) exhibited herpangina, and 10 (90.9 %) cases exhibited mild fever (<38.5 °C). There were no severe cases and no patients had serious complications.

### Viral pathogens of this outbreak

Four pharyngeal swab samples and three stool samples which were from six sick children and one close contact were positive for enterovirus, but negative for EV-A71 and CV-A16. The samples from the other close contacts, the surface of the classroom and other five sick children were negative to enterovirus with real-time PCR. Six fragments were amplified from two cases, one parent’s pharyngeal swab, and three stool samples. Sequences were genotyped as CV-A6 using NCBI BLAST and phylogenetic analyses.

### Nucleotide sequence similarity and phylogenetic analysis

**Fig. 1:**
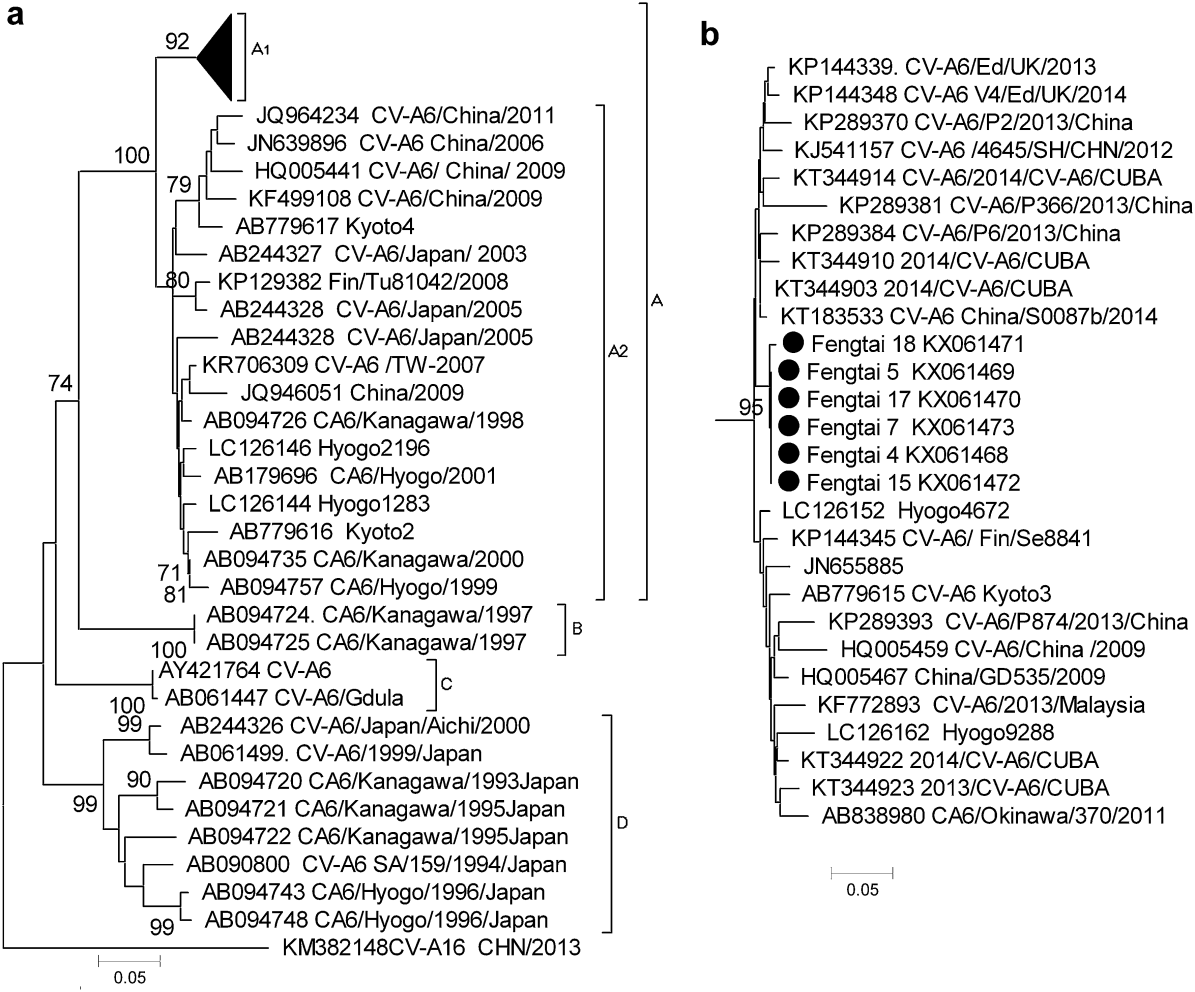
Phylogenetic analysis of CV-A6 complete VP4 nucleotide sequences phylogenetic analysis was performed and the tree was constructed using the neighbor-joining algorithm implemented in the MEGA version 5.0 software program with 1000 bootstrap pseudoreplicates. The numbers on the branches indicate the bootstrap values, excluding those <70 % for clarity. CV-A16 (accession no. KM382148.1) was used as an outgroup control. Black dots show CV-A6 strains isolated in this study. GenBank numbers of reference sequences are shown. **a** Phylogenetic analysis of the most reference strains of CV-A6. **b** Phylogenetic analysis of the subtree A1

Six CV-A6 clinical isolates shared 99–100 % and 100 % nucleotide and amino acid homology with other clinical isolates in China after 2013, respectively, but only 82 and 84 % homology to CV-A6 prototype strain Gdula. Four distinct lineages (ABCD) were revealed by phylogenetic analysis; lineage A was divided into two sublineages A1 and A2 (Fig. 1).

The phylogenetic analysis showed CV-A6 had continuously evolved and spread to other areas. In lineage A, most strains of sublineage A2 were detected in Japan before 2009 and a few from China and Finland, whereas sublineage A1 was a recent epidemic strain that first appeared in 2008–2009. CV-A6 was detected in more areas with a detection rate that markedly increased in the past 2 years. In Japan, lineages A, B and D were isolated at different years, however, lineages B and D were only isolated in Japan before 2000. Lineage B comprised strains isolated in 1997, whereas sublineage A2 was isolated in Japan before 2003 and then detected in other areas in 2008–2011. Only two strains of prototype strain Gdula from the United States and Japan comprised lineage C. Strains from this study clustered with epidemic strains isolated in 2013–2014 from China and other areas comprising sublineage A1.

### Control measures of the outbreak

CV-A6 is primarily transmitted via the fecal–oral route by contaminated hands, toys, or food. After the Fengtai Center for Disease Control and Prevention confirmed the outbreak with the China Information System for Disease Control and Prevention on July 19, normal control measures were taken, such as hand washing and classroom disinfection. Health education was also intensified in this Juku. Classes in the preschool art training department were suspended for 14 days starting July 21, and the children were continuously monitored for any new cases. No new case was reported up to August 7, indicating that the outbreak had ended.

## Discussion

HFMD outbreaks caused by enteroviruses except for EV-A71 and CV-A16 have recently increased worldwide (Osterback et al. 2009; Wei et al. 2011). Sporadic cases of CV-A6 infection are becoming more frequent, especially in China (Han et al. 2014; Lu et al. 2014; Hu et al. 2015; Bian et al. 2015). It was also recently reported as the major etiological agent in Changchun, north China. In 2013, CV-A6 replaced EV-A71 and CV-A16 as the main causative agent of HFMD, with a detection rate of 43.1 %, in the suburban areas of Beijing. (Han et al. 2014; Hongyan et al. 2014). This is the first HFMD outbreak caused by CV-A6 in Fengtai District in the past few years. But we have no the data of CV-A6 associated with the sporadic cases in Fengtai District. So more enterovirus need to be monitored in the HFMD cases.

Following the approval and application of EV-A71 vaccines in China, the etiological agents of HFMD are anticipated to change significantly in Fengtai District. Further investigation is needed to clarify significant changes in HFMD etiological agents other than EV-A71 and CV-A16. Our phylogenetic analysis of the CV-A6 also show that the virus is undergoing rapid evolution in recent years, becoming the main pathogen of HFMD, and spreading to other places where without the CV-A6 report. Thus, a more comprehensive monitoring system for HMFD and CV-A6 is needed to understand the evolution of CV-A6.

China Information System for Disease Control and Prevention is a powerful tool to detect outbreaks, and additional infectious diseases should be included in this system to implement early intervention measures for epidemics or outbreaks. Clinicians should strengthen the training to report the cases of infectious diseases, so that the control measures can be carried out at the early stage of the outbreaks which are critical to end the outbreaks as early as possible.

## Conclusions

This HFMD outbreak in a juku in Beijing, China, was caused by CV-A6. Measures, such as a sensitive surveillance system, early identification of the pathogen and timely intervention, are keys to controlling outbreaks of disease caused by CV-A6. To control the outbreak at the early stage, more types of diseases should be monitored by China Information System for Disease Control and Prevention.

## Nucleotide sequence accession numbers

Partial sequences of CV-A6 have been deposited under accession numbers: KX061468–KX061473.
